# Protein arginine methyltransferases PRMT1, PRMT4/CARM1 and PRMT5 have distinct functions in control of osteoblast differentiation

**DOI:** 10.1016/j.bonr.2023.101704

**Published:** 2023-07-25

**Authors:** Parisa Dashti, Eric A. Lewallen, Jonathan A.R. Gordon, Martin A. Montecino, Johannes P.T.M. van Leeuwen, Gary S. Stein, Bram C.J. van der Eerden, James R. Davie, Andre J. van Wijnen

**Affiliations:** a Department of Internal Medicine, Erasmus MC, Erasmus University Medical Center, Rotterdam, Netherlands; b Department of Orthopedic Surgery, Mayo Clinic, Rochester, MN, USA; c Department of Biological Sciences, Hampton University, Hampton, VA, USA; d Department of Biochemistry, University of Vermont, Burlington, VT, USA; e Institute of Biomedical Sciences, Faculty of Medicine, Universidad Andres Bello, Santiago, Chile; f Department of Biochemistry and Medical Genetics, University of Manitoba, Winnipeg, Manitoba R3E 0J9, Canada; g CancerCare Manitoba Research Institute, CancerCare Manitoba, Winnipeg, Manitoba R3E 0V9, Canada

**Keywords:** Bone, Osteoblast, Osteogenesis, Epigenetics, Chromatin, Histone, Mineralization

## Abstract

Osteogenic differentiation of mesenchymal cells is controlled by epigenetic enzymes that regulate post-translational modifications of histones. Compared to acetyl or methyltransferases, the physiological functions of protein arginine methyltransferases (PRMTs) in osteoblast differentiation remain minimally understood. Therefore, we surveyed the expression and function of all nine mammalian PRMT members during osteoblast differentiation. RNA-seq gene expression profiling shows that *Prmt1, Prmt4/Carm1* and *Prmt5* represent the most prominently expressed PRMT subtypes in mouse calvarial bone and MC3T3 osteoblasts as well as human musculoskeletal tissues and mesenchymal stromal cells (MSCs). Based on effects of siRNA depletion, it appears that PRMT members have different functional effects: (i) loss of *Prmt1* stimulates and (ii) loss of *Prmt5* decreases calcium deposition of mouse MC3T3 osteoblasts, while (iii) loss of *Carm1* is inconsequential for calcium deposition. Decreased *Prmt5* suppresses expression of multiple genes involved in mineralization (e.g., *Alpl*, *Ibsp*, *Phospho1*) consistent with a positive role in osteogenesis. Depletion of *Prmt1, Carm1 and Prmt5* has intricate but modest time-dependent effects on the expression of a panel of osteoblast differentiation and proliferation markers but does not change mRNA levels for select epigenetic regulators (e.g., *Ezh1*, *Ezh2*, *Brd2* and *Brd4*). Treatment with the Class I PRMT inhibitor GSK715 enhances extracellular matrix mineralization of MC3T3 cells, while blocking formation of H3R17me2a but not H4R3me2a marks. In sum, *Prmt1, Carm1 and Prmt5* have distinct biological roles during osteoblast differentiation, and different types histone H3 and H4 arginine methylation may contribute to the chromatin landscape during osteoblast differentiation.

## Highlights

• Protein arginine methyltransferases (PRMT) were surveyed for expression and functions during osteoblast differentiation.
• *Prmt1, Prmt4/Carm1* and *Prmt5* are prominently expressed in osteogenic cells.
• SiRNAs for *Prmt* proteins have different effects on calcium deposition and/or osteoblast marker gene expression.
• The PRMT inhibitor GSK715 enhances calcium deposition in MC3T3 osteoblasts.
• The results suggest that histone arginine methylation contributes to osteoblast differentiation.

## Graphical abstract

**Figure:**
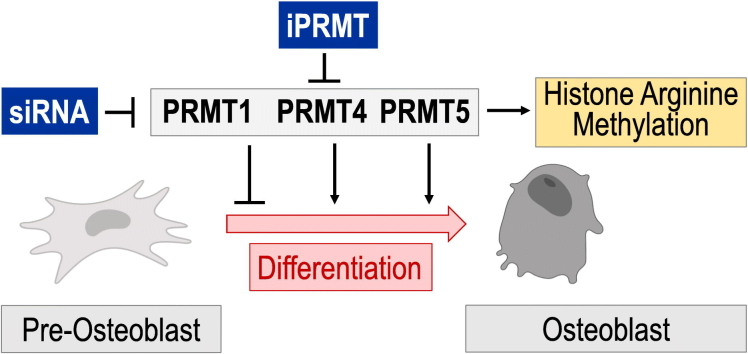

## 1 Introduction

Osteoblast differentiation is essential for bone formation and is controlled by intricate epigenetic mechanisms involving chromatin and non-coding RNA related processes that regulate the expression of bone related genes (Dudakovic et al., 2022; Gordon et al., 2015; Tai et al., 2014; van Wijnen et al., 2013; van Wijnen and Westendorf, 2019). Chromatin organization is dynamically altered by histone post-translational modifications (PTMs) that mediate epigenetic regulation of osteogenic lineage differentiation and osteoblast maturation (van Wijnen and Westendorf, 2019). We and others have identified multiple enzymes expressed in osteogenic cells that mediate or recognize lysine methylation (Dashti et al., 2023; Dudakovic et al., 2016; Dudakovic et al., 2020; Galvan et al., 2021; Khani et al., 2017; Moena et al., 2020a; Rojas et al., 2019; van Wijnen et al., 2021), demethylation (Dudakovic et al., 2017; Rojas et al., 2015; Rummukainen et al., 2022) or acetylation of histones (Dudakovic et al., 2013; Galea et al., 2020; Paradise et al., 2020; Paradise et al., 2022; Shen et al., 2003; Sierra et al., 2003), as well as DNA methylation and hydroxylation (Thaler et al., 2022). These epigenetic regulatory enzymes can induce modifications on different amino acids located on histone tails including lysines (K), arginines (R), and serines (S) (Tan et al., 2011). A subset of these writer, eraser and reader enzymes modifies histones on lysines to produce active (H3K4me3) or repressive marks (e.g., H3K27me3, H3K9me3) that modulate the accessibility of chromatin to transcription factors (van Wijnen and Westendorf, 2019; Delcuve et al., 2009). Both lysine methyltransferases (KMTs) and protein arginine methyltransferases (PRMTs) contribute to chromatin remodeling and osteoblast differentiation (Moena et al., 2020a; Beacon et al., 2021; Qian et al., 2018).

The protein arginine methyltransferase (PRMT) family is comprised of nine evolutionarily conserved proteins that catalyze the methylation of arginine residues (Kleinschmidt et al., 2008). These enzymes have broad protein substrates ranging from histones to non-histone nuclear and cytoplasmic proteins that differ in their activity by catalyzing asymmetrical arginine methylation (Class I; e.g., PRMT1 and 4), symmetrical arginine methylation (Class II; e.g., PRMT5) or arginine monomethylation (Class III; e.g., PRMT7). Each enzyme also has selectivity for different arginine moieties in histones H3 and H4, most of which represent gene activating marks. For example, PRMT1 asymmetrically catalyzes the formation of H4R3me2a (active mark), PRMT4/CARM1 catalyzes the formation of H3R17me2a (active mark), PRMT5 catalyzes the formation of symmetrical H3R2me2s (active mark), while PRMTs also mediate methylation of other less characterized histone marks (e.g., H3R26me2a, and H3R42me2a by PRMT4/CARM1) (Beacon et al., 2020; Jahan and Davie, 2015; Wu and Xu, 2012).

Methylation of arginine residues is fundamental for chromatin remodeling to support different cellular processes (e.g., RNA splicing, transcription, signal transduction, DNA damage response, and formation of phase separated domains) (Guccione and Richard, 2019; Min et al., 2019). Multiple PRMT family members have been shown to contribute to cell fate determination and metabolic pathways associated with human diseases, as well as mediate chromatin organization and function in different biological contexts (Jahan and Davie, 2015). Aberrant expression of PRMTs is correlated with some cancers (Kim and Ronai, 2020), including osteosarcoma (Li et al., 2017). PRMT1, PRMT4/CARM1, PRMT5 and PRMT7 are prevalent isoforms that are functionally expressed in myogenic cells and osteosarcoma cells (Moena et al., 2020a; Blanc and Richard, 2017; Rakow et al., 2020). Three PRMTs (i.e., PRMT1, PRMT4/CARM1 and PRMT5) have each been shown to control vitamin D3-dependent transcription in osteosarcoma cells via interactions with SRC-1/NCOA1 (Moena et al., 2020b), whereas PRMT5 binds to the SWI/SNF component BRG1/SMARCA4 in different cell types (Seth-Vollenweider et al., 2014). These findings collectively suggest that PRMT proteins may also have a role in osteoblast differentiation. However, there are no studies that have comprehensively analyzed PRMT expression and function during MC3T3 osteoblast differentiation that would permit a specific focus on a key PRMT protein.

Because PRMTs have broad biological functions, a key question in bone biology is whether and how PRMT family members control osteogenic differentiation of mesenchymal stem/stromal cells (MSCs) and osteoblasts, as well as bone formation and bone homeostasis (i.e., osteoblast and osteoclast interplay). Initial studies have suggested individual roles of PRMTs in musculoskeletal tissues, including skeletal myoblast differentiation, chondrocyte proliferation and osteoblastic cells (Moena et al., 2020b; Seth-Vollenweider et al., 2014; Ito et al., 2009). In this study we surveyed the expression and functional contributions of multiple PRMTs to osteoblast differentiation. The results show that three distinct PRMTs (PRMT1, PRMT4 and PRMT5) regulate early stages of osteoblast differentiation by either enhancing or inhibiting osteoblast related gene expression and calcium deposition. Furthermore, selective pharmacological inhibition of PRMTs can enhance osteoblastogenesis.

## 2 Materials & methods

### 2.1 High throughput RNA sequencing and bioinformatic analysis

RNA-seq data was retrieved from human musculoskeletal tissues including bone, cartilage, growth plate, muscle and adipose tissue, as previously reported (Paradise et al., 2018). In addition, we included RNA data from bone marrow derived MSCs from two different healthy donors that were subjected to osteogenic differentiation for fifteen days. In addition, we included data from mouse callus (Khani et al., 2017; Matthews et al., 2014), as well as undifferentiated mouse MC3T3 osteoblasts, ATDC5 chondrocytes and C2C12 cells that were cultured in their respective maintenance media (Dashti et al., 2023). High throughput mRNA sequencing and bioinformatic analyses were performed as previously reported (Dudakovic et al., 2014) with mRNA levels expressed as fragments per kilobase pair per million mapped reads (FPKM).

### 2.2 Cell culture

Our studies used MC3T3 E1 sc4 murine calvarial osteoblasts [PMID: 10352097] that were acquired from the American Type Culture Collection. These cells were cultured at 37 °C in 5 % CO_2_ with complete maintenance medium, which is α-minimal essential medium (Gibco) without ascorbic acid and supplemented with 10 % fetal bovine serum (Atlanta Biologicals), 100 units/ml penicillin (Gibco), and 100 μg/ml streptomycin (Gibco). MC3T3 cells were seeded in 6- or 12-well plates in maintenance medium (10,000 cells/cm^2^). The following day (day 0), we divided the cultures into two groups. Cells were subjected to siRNA transfections with control (D-001810-10-20; GE Healthcare, Chicago, IL), Prmt1 siRNA (Mouse *Prmt1* ON-TARGET*plus* siRNA SMARTpools; L-049497-00-0005; GE Healthcare), *Prmt4/Carm1* siRNA (Mouse Carm1 ON-TARGET*plus* siRNA SMARTpools; L-048766-00-0005; GE Healthcare), *Prmt5* siRNA (Mouse Prmt5 ON-TARGET*plus* siRNA SMARTpools; L-042281-00-0005; GE Healthcare). Transfections were performed using RNAiMAX as instructed by the manufacturer (Invitrogen). After 6 h, osteogenic medium consisting of complete maintenance medium supplemented with 50 μg/ml ascorbic acid (Sigma) and 4 mM β-glycerol phosphate (Sigma) was added, and the cells were cultured until harvest. Studies with the PRMT inhibitor GSK715 were performed similarly, except that instead of transfection, cells were treated with GSK715 one day after seeding (day 0; 1× treatment) or also after three days (day 0 and day 3; 2× treatment). For both siRNA and PRMT inhibitor studies, we selected the time of harvest based on the first visual appearance of calcified extracellular matrix nodules which typically occurs between days 21 and 32 depending on the passage number, actual seeding density and type of experimental treatment.

### 2.3 Quantitative real-time reverse transcriptase PCR (RT-qPCR)

Cells were lysed using TRIzol Reagent (Invitrogen) and RNA was isolated using the Direct-zol RNA MiniPrep Kit (Zymo Research) with on column DNA digestion kit (Zymo Research). Isolated RNA was reverse transcribed into cDNA using the SuperScript III First Strand Synthesis System (Invitrogen). Gene expression was quantified using real-time PCR in which each reaction was performed with 10 ng cDNA per 10 μl, QuantiTect SYBR Green PCR Kit (Qiagen), and the CFX384 Real-Time System machine (BioRad). Transcript levels were quantified using the 2^−ΔΔCt^ method and normalized to the housekeeping gene *GAPDH/Gapdh* using gene specific primer sequences (Table 1).

**Table 1:** Summary of RT-qPCR primers used in this study. The mRNAs for the indicated mouse genes (left column) were amplified using gene specific forward primers (middle column) and reverse primers (right column).

RT-qPCR primers (5′ - 3′)		
Gene symbol	Forward	Reverse
Prmt1	CTGCCTCTTCTACGAGTCCATG	TCGGTCCTCAATGGCTGTCACA
Carm1	TCGAGAGCTACCTCCATGCCAA	GGCTTTGGTGAACTGCTCCATG
Prmt5	CCTGCTTTACCTTCAGCCATCC	GCACAGTCTCAAAGTAGCCTGC
Sp7	GAGGCAACTGGCTAGGTGG	CTGGATTAAGGGGAGCAAAGTC
Runx2	CCTGAACTCTGCACCAAGTCCT	TCATCTGGCTCAGATAGGAGGG
Dlx5	AGGCTTATGCCGACTACGGCTA	CTCTGGCTCCGCCACTTCTTTC
Ibsp	GAATGGCCTGTGCTTTCTCG	CCCGTACTTAAAGACCCCGTT
Bglap	GCAATAAGGTAGTGAACAGACTCC	CCATAGATGCGTTTGTAGGCGG
Alpl	CCAGAAAGACACCTTGACTGTGG	TCTTGTCCGTGTCGCTCACCAT
Spp1	GCTTGGCTTATGGACTGAGGTC	CCTTAGACTCACCGCTCTTCATG
Phosphp1	TTATTCCGCCGCATCCTCAGCA	TCACGCAGGTATTCGCTGAGCA
Col1a1	CCTCAGGGTATTGCTGGACAAC	CAGAAGGACCTTGTTTGCCAGG
Ezh1	CGAGTCTTCCACGGCACCTATT	GCTCATCTGTTGGCAGCTTTAGG
Ezh2	CATACGCTCTTCTGTCGACGATG	ACACTGTGGTCCACAAGGCTTG
Brd2	CAATCCTCCAGACCACGATGTTG	TGGTAGAGGTCCTGGTTCCAGT
Brd4	GCCATCTACACTACGAGAGTTGG	ATTCGCTGGTGCTCTCCGACTC
Ccnb2	GCACTACCATCCTTCTCAGGTG	TGTGCTGCATGACTTCCAGGAC
Mki67	GAGGAGAAACGCCAACCAAGAG	TTTGTCCTCGGTGGCGTTATCC
Gapdh	CATCACTGCCACCCAGAAGACTG	ATGCCAGTGACTTCCCGTTCAG

### 2.4 Western blotting

MC3T3-E1 cells (10,000 cells/cm^2^) were plated in 6-well plates in maintenance medium and treated with siRNA (see above) or the Class I PRMT inhibitor GSK715 (2 and 5 μM final concentration from a 10 mM stock solution in DMSO; GSK3368715 or EPZ019997, MedChemExpress, Monmouth Junction, NJ) as described previously (Fedoriw et al., 2019). Cells were lysed in radioimmunoprecipitation buffer (150 mM NaCl, 50 mM Tris, pH 7.4, 1 % sodium deoxycholate, 0.1 % sodium dodecyl sulfate, 1 % Triton X-100) supplemented with protease inhibitor mixture (Sigma) and phenylmethylsulfonyl fluoride (Sigma). Lysates were cleared by centrifugation. Protein concentrations were determined by the DC Protein Assay (Bio-Rad). Proteins were resolved by SDS-PAGE and transferred to polyvinylidene difluoride membranes. After blocking in 5 % nonfat dry milk for 90 min at room temperature, primary antibodies were added overnight at 4 °C, followed by secondary antibodies for 1 h at room temperature. Proteins were visualized using an ECL Prime detection kit. The following primary antibodies were used: PRMT1(F339) (1:1000; 2453S; Cell Signaling), PRMT4/carm1 (1:1000; 4438S; Cell Signaling), PRMT5(D5P2T) (1:1000; 79998S; Cell Signaling), H4R3me2a (1:1000; PA5-102612; Invitrogen) and H3 (1:10,000; 05-928; Millipore).

### 2.5 Alizarin Red staining

To assess calcium deposition in the extracellular matrix of MC3T3 cultures, we fixed cells in 10 % neutral buffered formalin for 20 min and stained with 2 % Alizarin Red (Sigma) for 12 min. Upon removal of the Alizarin Red, plates were rinsed for 3 times with distilled water and imaged using a standard plate scanner. Absorption of Alizarin Red stain was quantified with ImageJ software (Dudakovic et al., 2020). Because Alizarin Red staining is saturable, we harvested samples well before they reach maximal staining. We note that cultures were visually inspected for the first appearance of bone-like nodules in either the control or test samples (treated with siRNA or GSK715), and harvested simultaneously at a time that aims to maximize differences between control and the specific treatment. For this reason, there are differences in the extent of calcium deposition in the control samples in distinct experiments, because they were harvested on different days.

### 2.6 Statistics

Statistical analysis and generation of figures was performed with GraphPad Prism software (version 9) using Student's unpaired *t-*test or two-way ANOVA for multiple comparison analyses. Histochemical assays and RT-qPCR time course experiments were performed in triplicate for each time point; each RT-qPCR measurement was performed as a technical duplicate. Experiments were replicated three times and results from a representative course are shown. Significance is denoted by asterisks in the figures (p < 0.05, *; p < 0.01, **).

## 3 Results

### 3.1 Selective expression of PRMT genes in mouse osteoblasts and human bone tissues

**Fig. 1:**
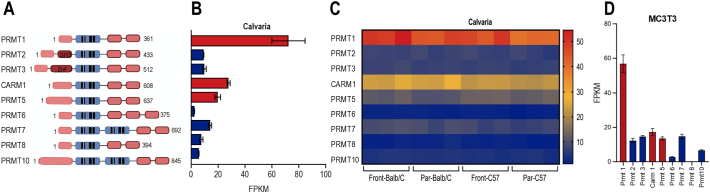
Expression of multiple *Prmt* genes in craniofacial tissue and MC3T3 cells. **(A)** Structure of PRMT family member proteins. The diagram shows the domain structure of the nine PRMT members expressed in human and mouse. **(B)** RNA-seq data show expression levels (in FPKM) revealing that *PRMT1* has the highest expression among nine *Prmt* mRNA levels in wild type mouse calvaria, while *Prmt4/Carm1* and *Prmt5* are the next two highly expressed PRMT members, respectively. Graphs include n = 3 samples and show mean + STD. **(C)** Heatmap of mRNAs for *Prmt* family members by RNA-seq from frontal and parietal calvaria in Balb/C or C57/B6 mice. Graphs include n = 3 samples for each sample group and show mean + STD. **(D)** RNA-seq data shows that *PRMT1* has highest expression and, *PRMT4/CARM1* and *PRMT5* are also expressed in mouse MC3T3 cells (n = 6 samples, mean + STD).

**Fig. 2:**
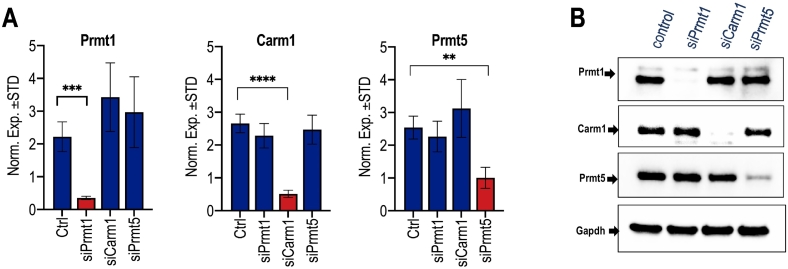
*PRMT* loss of function validation by RT-qPCR and western blotting in differentiating MC3T3 cells. RNAs and proteins were isolated on day 3 to validate *Prmt* knock-down by RT-qPCR and western blotting. **(A)** Parallel RT-qPCR analysis for *Prmt* mRNAs (*Prmt1*, *Prmt4/Carm1*, *Prmt5*) on day 3 of differentiation (n = 3, mean ± STD; ** = p < 0.01, *** = p < 0.001, and **** = p < 0.0001). (**B**) Western blotting analysis for PRMT1, PRMT4/CARM1, PRMT5 and GAPDH was performed on day 3 of differentiation.

The PRMT family of methyl arginine transferases has been conserved during mammalian genome evolution, reflecting the important contributions of these epigenetic regulators to embryonic development and epigenetic regulation. There are nine different PRMT isoforms which include Class I, II and III enzymes, with distinct protein domains; the variations in protein structure may have evolved to accommodate specific epigenetic functions in different tissues (Fig. 1A). We performed expression profiling using RNA-seq datasets from our previous studies to understand where and when human *PRMT* or mouse *Prmt* genes are expressed in musculoskeletal cell types and tissues. RNA-seq data from mesenchymal versus non-mesenchymal cell types in mouse callus during fracture repair show that *Prmt1*, *Prmt4/Carm1* and *Prmt5* are preferentially expressed in mesenchymal cell types compared to non-mesenchymal cell types in callus tissue (sorted using a dsTomato fluorescent protein lineage tracer) (Matthews et al., 2014) at day 2 after fracture in the callus site (Fig. S1). Additionally, the data show that multiple *PRMT* isoforms are expressed in adult human musculoskeletal tissues (Fig. S2). *PRMT1*, *PRMT4/CARM1* and *PRMT5* are the three most prominently expressed *PRMT* members across all tissues examined (i.e., bone, cartilage, muscle, growth plate and adipose tissue). Whereas *PRMT1* is the highest expressed member in each of the tissues, *PRMT4/CARM1* and *PRMT5* are particularly prominent in muscle and cartilage, respectively (Fig. S2). *PRMT* mRNAs were also examined in human bone marrow derived mesenchymal stem cells (BMSCs) (Fig. S3). Similar to expression in musculoskeletal tissues, we find that *PRMT1*, *PMRT4/CARM1* and *PRMT5* are the most highly expressed PRMT members in human BMSCs, and their expression remains relatively constant during osteogenic lineage differentiation (Fig. S3). Furthermore, *Prmt1*, *Prmt4* and *Prmt5* are also highly expressed in frontal and parietal bone of calvaria from two different mouse strains (Fig. 1B&C). Similarly, these three PRMT members are also robustly expressed in MC3T3 cells which represent immortalized osteoblast precursors derived from mouse calvaria (Fig. 1D). Expression of *Prmt1*, *Prmt4/Carm1* and *Prmt5* mRNAs and proteins in MC3T3 cells was confirmed by RT-qPCR and western blot analysis (Fig. 2). Taken together, these data suggest that *PRMT1/Prmt1*, *PMRT4/Prmt4* and *PRMT5/Prmt5* are selectively expressed in both human and mouse musculoskeletal tissues. Because these three mRNAs are generally more prevalently expressed than other PRMTs, we prioritized these three genes for loss of function studies during osteoblastic differentiation.

### 3.2 Selective modulation of osteoblast differentiation upon siRNA depletion of *PRMT1*, *PMRT4/CARM1* and *PRMT5*

**Fig. 3:**
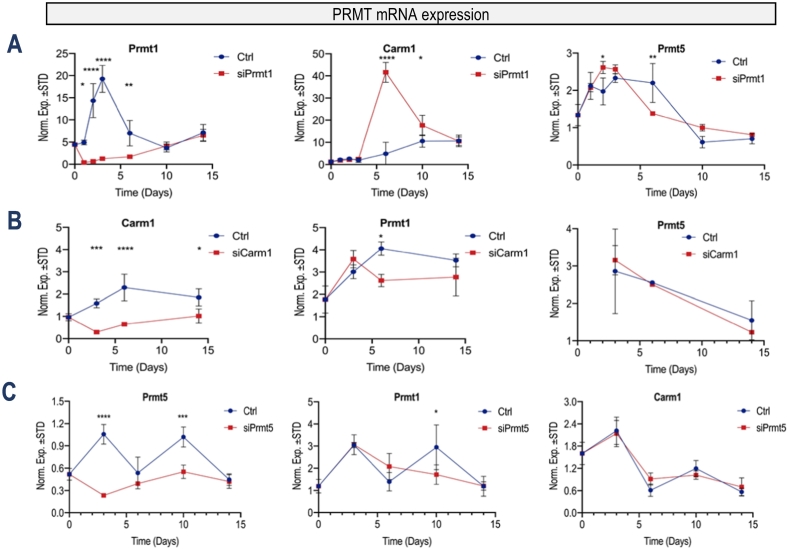
*PRMT* knock-down effects on mRNA levels of other *PRMT* members during osteoblast differentiation. Time course expression analysis using RT-qPCR shows that siRNA depletion since day 0 suppresses select *Prmt* mRNA levels. **(A)***Prmt4/Carm1* mRNA levels are enhanced upon *Prmt1* knock-down at day 6 after induction of osteoblast differentiation. **(B)***Prmt1* and *Prmt5* mRNA levels do not show dramatic change after *Prmt4/Carm1* loss of function. **(C)***Prmt1* and *Prmt4/Carm1* mRNA levels do not show appreciable modulations in mRNA levels upon PRMT5 loss of function over the osteoblast differentiation in 15 days. (n = 3, mean ± STD; ** = p < 0.01, *** = p < 0.001, and **** = p < 0.0001).

To investigate the functional contributions of *Prmt1*, *Prmt4/Carm1* and *Prmt5* in mouse osteoblastogenesis, we conducted loss of function studies using siRNAs in MC3T3 cells. We examined short-term effects of siRNA depletion at both mRNA and protein levels (Fig. 2), as well as longer-term effects during a 2 week time-course (Fig. 3). To establish specificity of the siRNA knockdown, we also analyzed effects of each siRNA on the expression of the other two PRMT members, while also assessing the potential for cross-regulation by different PRMT subtypes. Transfection of cells with commercial siRNA pools results in specific knockdown of all three genes at both the mRNA- and protein-levels three days after siRNA treatment, as assessed by RT-qPCR (Fig. 2A) and western blot analysis (Fig. 2B). Time-course analysis during MC3T3 osteoblast differentiation reveals that siRNA treatment during the initial 3 days of the culturing period dramatically alters the temporal expression of each *Prmt* isoform and expression returns to levels observed in control cells treated with non-silencing RNA after 10 to 14 days (Fig. 3). Hence, the siRNA depletion for each *Prmt* gene remains effective during the first week of the culturing period.

To understand whether depletion of one *Prmt* member affects the expression of the other two *Prmt* members, we examined the expression of all three members concurrently in response to siRNA depletion. We find that siRNAs for any *Prmt* member do not affect the expression of other *Prmt* members during the first three days of the culturing period as is expected (Fig. 2, Fig. 3). Remarkably and unexpectedly, knockdown of *Prmt1* results in a transient upregulation of *Prmt4/Carm1* but not *Prmt 5* at later cell culture stages (i.e., at day 7 and day 10) (Fig. 3). *Prmt4/Carm1* depletion appears to have a negative effect on *Prmt1* levels on day 6, but the magnitude of this effect is marginal (Fig. 3). Collectively, these results suggest that siRNA knockdown of *Prmt* members may have effects on *Prmt* mRNA expression for 7 to 10 days. The results also suggest that interpretations of *Prmt1* function after mRNA depletion may be confounded by a compensatory increase in mRNA levels of *Prmt4/Carm1*. The latter could potentially substitute for *Prmt1* during MC3T3 osteoblast differentiation.

**Fig. 4:**
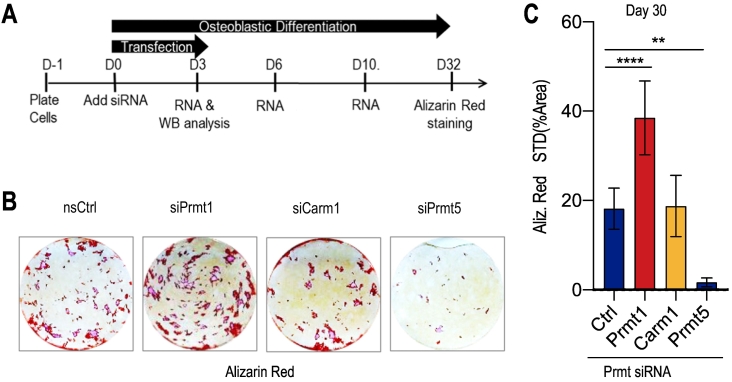
Loss of *Prmt1, Prmt4/Carm1, and Prmt5* upon siRNA transfection has different effects on calcium deposition of MC3T3 cells. **(A)** A time course of osteoblast differentiation shows upregulation of **(B)** representative Alizarin Red stained cultures. The results show that MC3T3 cell mineralization is enhanced upon *siPrmt1* treatment, remains the same as control upon *siCarm1* treatment, and is decreased after *siPrmt5* knock-down. **(C)** Quantification of mineralization in differentiating cells on day 30 using Image J software. Statistical significance was determined by two-way ANOVA (n = 3, mean + STD; * = p < 0.05 and **** = p < 0.0001). We note that for each of the siRNAs, we stained the cultures as pairs of nsRNA vs siRNA at different times when mineralization would be apparent in either the control or treatment group; hence, mineralization in controls differs for each of nsRNA/siRNA pairs (respectively, for PRMT1, CARM1 and PRMT5). The time course endpoint of Alizarin Red staining is determined by the appearance of visual evidence for nodular calcium deposition and varies between experiments (day 21 to day 32).

**Fig. 5:**
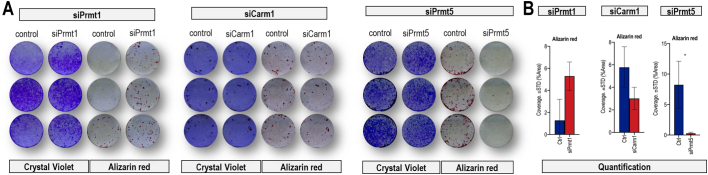
Crystal violet staining validation for mineralization of MC3T3 cells upon *siPrmt1*, *siCarm1*, and *siPrmt5* transfection. **(A)** Crystal violet staining was carried out in mineralized MC3T3 cells on day 30 reflecting total cell accumulation in each well (3 biological replicates) to indicate that MC3T3 mineralization is independent of cell numbers. **(B)** Quantification of mineralization in differentiating cells on day 30 using Image J software. Statistical significance was determined by two-way ANOVA (n = 3, mean + STD; * = p < 0.05). The time course endpoint of Alizarin Red staining is determined by the appearance of visual evidence for nodular calcium deposition and varies between experiments (day 21 to day 32).

We assessed whether loss of *Prmt1*, *Prmt4/Carm1* or *Prmt5* affects calcium deposition using Alizarin Red staining (Fig. 4, Fig. 5). Calcium accumulation is evident for control cells (treated with nsRNA) at day 30 of the cell culturing period (Fig. 4). Loss of *Prmt1* increases calcium deposition (∼2 fold increase; p < 0.001), while loss of *Prmt4/Carm1* has no appreciable effects on calcium accumulation. In contrast, inhibition of *Prmt5* dramatically decreases calcium deposition (∼10-fold reduction; p < 0.01). There is no clear correlation between mineralization and Crystal Violet staining (Fig. 5), but the presence of Crystal Violet staining across each culture dish indicates that the absence of Alizarin Red staining is not simply due to local loss of cells. Taken together, all three *Prmt* members have different roles in osteoblast differentiation: *Prmt1* inhibits and *Prmt5* is required for osteoblast differentiation, while *Prmt4/Carm1* loss by itself is dispensable for calcium deposition by osteoblasts. The finding that *Prmt1* inhibits osteoblast differentiation is in agreement with other studies showing that this protein promotes the undifferentiated state of cells (Goruppi et al., 2023).

### 3.3 Loss of *Prmt1*, *Prmt4/Carm1* and *Prmt5* alters expression of stage-specific mRNA biomarkers for osteoblast differentiation

To understand whether depletion of PRMT proteins between day 0 and day 4 affects proliferation-related or osteoblast-specific gene expression, we examined cell cycle regulated genes (i.e., Cyclin B*/Ccnb1* and Ki-67/*Mki67*) (Fig. S4). RT-qPCR analysis shows that siRNA depletion of each PRMT protein does not have strong numerical effects on cell cycle regulated mRNAs, including expression of the mitosis-related mRNA marker *Ccnb1* or the proliferation-related *Mki67* mRNA (Fig. S4). We note that expression values at some time points exhibit statistical significance. However, the observed numerical changes cannot easily be interpreted and may not necessarily be biologically relevant. At face value, loss of CARM1 at day 7 reduces the mitosis related marker CCNB2 and increases the general proliferation marker MKI67. The latter would suggest that absence of CARM1 may decrease the G2/M population and increase the number of cells in G1 phase around day 7. Because loss of CARM1 does not alter Alizarin Red staining, these molecular changes have uncertain biological relevance. Furthermore, loss of PRMT5 results in a significant albeit quantitatively modest reduction (<2 fold) of both CCNB2 and MKI67, suggesting that PRMT5 loss may induce a quiescent cell state. The latter could potentially contribute to a reduced level of calcium deposition by preventing confluence of MC3T3 cell cultures (see Fig. 4). Crystal Violet data show qualitatively that cells are distributed across the entire cell culture plate, indicating that differences in Alizarin Red staining are not due to the complete absence of cells (Fig. 5). We conclude that effects of PRMT loss on cell proliferation or quiescence are not sufficiently dominant to account for changes in calcium deposition but may contribute to changes in Alizarin Red staining.

**Fig. 6:**
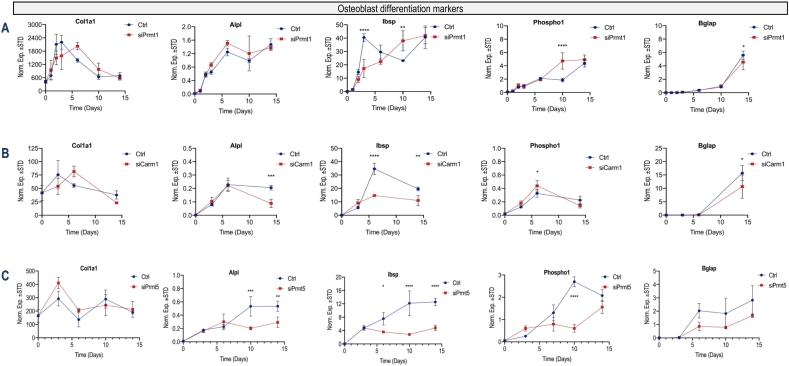
*Prmt1 loss* stimulates and *Prmt5* loss inhibits osteogenic differentiation markers of MC3T3 cells. RT-qPCR analysis for osteogenic mRNA markers (*Col1a1*, *Alpl*, *Ibsp*, *Phosphpo1*, and *Bglap*) after knockdown with **(A)***Prmt1*, **(B)***Prmt4/Carm1*, **(C)***Prmt5* during a 15 day time course of MC3T3 differentiation (n = 3, mean ± STD; ** = p < 0.01, *** = p < 0.001, and **** = p < 0.0001). Because effects of PRMT depletion are evident at day 14, we did not examine mRNA expression beyond this time point.

Although siRNAs typically are effective for up to four days, our data show that siPRMT1, siCARM1/PRMT4 an siPRMT5 effectively decrease the mRNA expression of the corresponding PRMT members for about one week. We extended this time period of analysis to 14 days to examine the recovery of MC3T3 cells after siRNA depletion and to assess effects on mature osteoblast markers (e.g., *Col1a1*, *Alpl*, *Ibsp*, *Phospho1* and *Bglap*) which are typically expressed at maximal levels around day 14 (Fig. 6). Calcium deposition was analyzed by Alizarin Red (see Fig. 4, Fig. 5) in separate cultures because this process requires a much longer time (up to 32 days); we did not directly align our mRNA analyses with histochemical examination of calcium deposition. The expression of osteoblast markers during osteoblast differentiation follows a well-established temporal sequence of, first, expression of collagenous (*Col1a1*) and non-collagenous (*Ibsp*) genes and, second, of expression of mineralization specific genes (*Bglap* and *Phospho1*). Reduced *Prmt1* expression significantly increases expression of *Ibsp* and *Phospho1* at day 10 (p < 0.01), but does not appear to have statistically significant effects expression of *Alpl* or *Bglap* at any timepoint. Interestingly, *Ibsp* expression is reduced by *Prmt1* loss at day 3 (Fig. 5). Hence, the effects of *Prmt1* loss could be biphasic and/or time-dependent. From a temporal perspective, time course curves can be condensed or stretched to reflect acceleration or deceleration of osteoblast differentiation. From this temporal perspective, evaluation of the entire mRNA expression time course upon *Prmt1* depletion shows that *Ibsp* expression is modestly delayed (at day 3; p < 0.001) while *Phosho1* expression may be accelerated at day 10 (Fig. 6). Diminished expression of *Prmt4/Carm1* correlates with significantly decreased expression of *Ibsp* and *Phospho1* at day 6, as well as decreased expression of *Alpl* and *Bglap* at day 14 (p < 0.01). While these changes indicate that loss of *Carm1/Prmt4* has effects on osteoblast-related gene expression (Fig. 6), the biological relevance of these expression changes is uncertain because loss of *Prmt4/Carm1* does not affect osteoblast mineralization (Fig. 4, Fig. 5). More interestingly, depletion of Prmt5 expression, which inhibits osteoblast mineralization (Fig. 4, Fig. 5) significantly decreases expression of *Alpl* and *Ibsp*, at days 10 and 14 (p < 0.01), and *Phospho1* at day 10, while there appears to be a time-course trend for decreased expression of *Bglap* at days 6, 10 and 15 (Fig. 6). Collectively, these gene expression data indicate that *Prmt1*, *Prmt4/Carm1* and *Prmt5* alter the expression of distinct osteoblast markers to different degrees and with distinct temporal dynamics.

Because PRMT1, PRMT4/CARM1 and PRMT5 proteins modulate distinct osteoblast markers at different stages of osteoblast differentiation, we assessed whether these epigenetic regulators may have indirect effects on osteoblastogenesis by controlling the mRNA expression of osteoblast related transcription factors (Fig. S5) or other epigenetic regulators (Fig. S6). The only remarkable finding of these RT-qPCR results is that *Prmt4/Carm1* loss reduces expression of *Runx2* mRNA (at day 6, p < 0.001), with a numerical trend of decreased expression for *Runx2*, *Osterix/Sp7* and *Dlx5* during the differentiation time-course (Fig. S5) that was not statistically validated. Aside from significant reduction of Sp7 in the siPrmt5 treatment group, expression analysis of these transcription factors upon depletion of *Prmt1* or *Prmt5* did not yield unambiguous results that permit a straightforward interpretation (Fig. S5C). Similarly, loss of *Prmt1, Carm1* and *Prmt5* mRNAs does not appear to modulate expression of epigenetic regulators known to contribute to skeletal development (e.g., Ezh2, Brd4) (Paradise et al., 2020; Dudakovic et al., 2018), except *Brd2* which is decreased by Prmt5 at an early stage of differentiation (day 3; Fig. S6). We conclude that PRMT1, PRMT4/CARM1and PRMT5 proteins have functional roles that are not directly rate-limiting for the expression of key osteoblast related transcription factors or other epigenetic regulators.

### 3.4 Pharmacological inhibition with the Class I PRMT inhibitor enhances calcium deposition in the extracellular matrix of MC3T3 cells

**Fig. 7:**
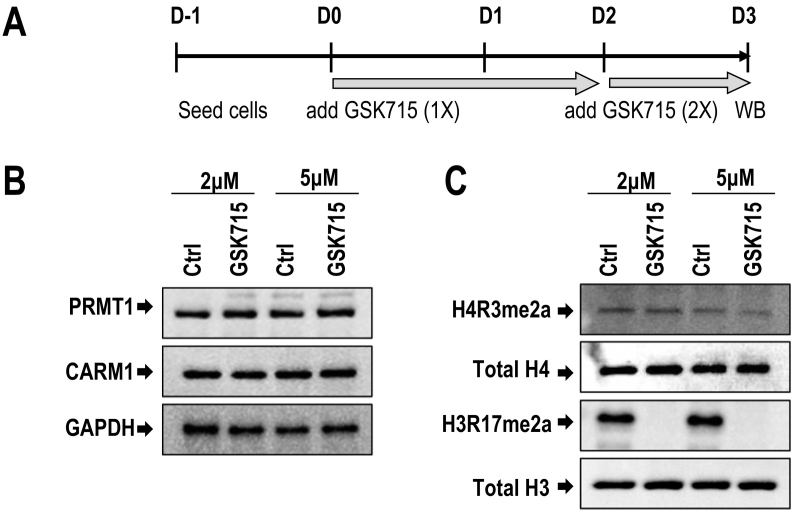
The small molecule inhibitor, GSK715, blocks H3R17me2a protein level after dual administration in MC3T3 cells. **(A)** MC3T3 cells were treated by GSK715 (Type 1 PRMT inhibitor) on day 0 and day 2 (2× treatment), and protein lysates were collected on day 3. **(B)** Western blotting shows PRMT1 and PRMT4/CARM1 protein levels do not change by dual administration of either 2 μM or 5 μM of GSK715 on day 3 compared to the GAPDH protein level. **(C)** Western blot analysis shows that GSK715 reduces levels of H3R17me2a (mediated by Pmrt1) but not H4R3me2a (which is mediated by PRMT4/CARM1). Administration of GSK715 at two different doses (2 μM and 5 μ) has no biological effects on total H4 or H3 protein levels (representative gel of 3 biological replicates).

To investigate whether the enzymatic activity of Class I PRMT enzymes (e.g., PRMT1, CARM1) affects osteoblastogenesis, we performed studies with the pharmacological inhibitor GSK715 (Fig. 7A). Treatment of MC3T3 osteoblasts with GSK715 reduces the nuclear levels of H3R17 asymmetric methylation (H3R17me2a), but not the levels of H4R3 asymmetric methylation (H4R3me2a) (Fig. 7B&C); the H3R17me2a versus H4R3me2a marks are generated by, respectively, PRMT4/CARM1 versus PRMT1. As expected, GSK715 also does not affect the protein levels of either H3 or H4, or the two Class I proteins (PRMT1 and CARM1) (Fig. 7B&C). These results indicate that GSK715 primarily affects nuclear levels of the CARM1 dependent methylation of H3R17me2a in MC3T3 cells.

**Fig. 8:**
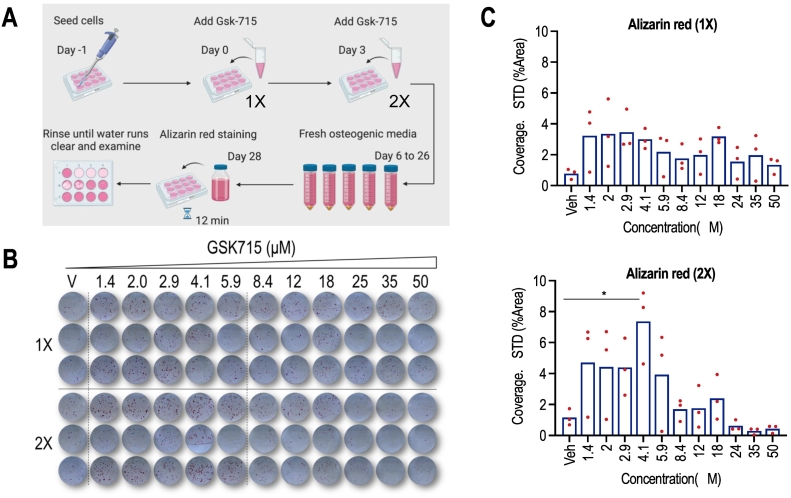
The small molecule inhibitor, GSK715, enhances MC3T3 mineralization in MC3T3 cells **(A)** Schematic illustration of MC3T3 showing GSK715 treatment over a range of concentrations (1 to 50 μM) and mineralization measurements after 28 days. **(B)** Alizarin red staining on day 28 of osteoblast differentiation shows that single (1×, day 0 to day 3) or dual administration (2×; from day 0 to day 3, and from day 3 to day 6) of 1 to 6 μM of GSK715 increases MC3T3 mineralization compared to control. **(C)** Quantification of mineralization in differentiating cells on day 28 using Image J software. Statistical significance was determined by two-way ANOVA (n = 3, mean + STD).

We tested the effects of GSK715 on MC3T3 differentiation using Alizarin Red staining (Fig. 8). MC3T3 cultures were treated with doses from 1 to 6 μM GSK715 that were either applied once (from day 0 to day 3, 1× dosing) or twice (from day 0 to day3 and day 3 to day 6; 2× dosing). In general, we find that a double dosing (i.e., drug administration repeated between day 3 and day 6) is more effective than single dosing (Fig. 8). Histochemical examination using Alizarin Red staining reveals that at least one of these regimens for GSK715 (6 μM administered twice) significantly enhances calcium deposition in MC3T3 osteoblast cultures as a proxy for osteoblast differentiation (Fig. 8). As the data overlays show, we observed a numerical trend of increased average values of Alizarin Red staining. However, this numerical trend was not matched by a statistical trend based on standard *t*-tests compared to controls. None of these observed changes, except for one, was statistically significant (p-values > 0.05) due to the large spread in values.

The densitometric values of Alizarin Red quantification for each of the dose response curves were also fitted to a polynomial curve (Fig. S7). The relative fit for the 2× dose response curve was better than the 1× dose response curve that have R^2^ values 0.5589 and 0.2185, respectively (Fig. S7A&B). Interestingly, the fitted lines for the 2× curve (which has an acceptable R^2^ value that clarifies >50 % of the observed variation in values) shows a consistent 3 to 6 fold increase in densitometric values for cells treated with concentrations of between 1.4 and 5.9 μM GSK175; for example, comparison of staining of cells treated with vehicle (0 μM; black dotted line) compared to a representative low dose of GSK175 (2.9 μM; red dashed line) reveals a 4.3 fold stimulation in Alizarin Red staining by GSK715 (Fig. S7B).

We note that GSK715 has biphasic effects on MC3T3 cells with apparent positive effects at low doses and negative effects of GSK715 at higher doses (Figs. 8 & S7). Negative effects of GSK715 at high micromolar concentrations are likely due to general cytotoxicity. In our prior experience with a large panel of EZH2 related epigenetic drugs (Galvan et al., 2021), we observed similar biphasic effects. Therefore, cytotoxic effects of high doses of a PRMT-related epigenetic drug on osteoblast differentiation are not unexpected.

Collectively, the findings of these pilot studies indicate that pharmacological inhibition of Class I PRMT enzymes that control nuclear H3R17me2 levels may increase calcium deposition during osteoblast differentiation. Comparison of findings obtained with GSK715 versus siRNA treatments for Prmt1, Prmt4 and Prmt5 indicates that none of these three epigenetic enzymes by themselves can account for the positive effect of the inhibitor on osteoblast mineralization. In conclusion, histone arginine methylation contributes to osteoblast differentiation and selective pharmacological inhibition of PRMTs may promote osteoblastogenesis.

## 4 Discussion

In this study, we examined the contribution of histone arginine methylation to osteoblast differentiation by surveying the expression and function of the full complement of human PRMT proteins. Our findings establish that *PRMT1*, *PRMT4/CARM1* and *PRMT5* mRNAs are selectively expressed in human and mouse musculoskeletal tissues. These three genes are generally most prominently expressed (i) across multiple human musculoskeletal tissues examined (i.e., bone, cartilage, muscle, growth plate and adipose tissue), (ii) in frontal and parietal bone of mouse calvaria, (iii) in human bone marrow derived MSCs and mouse MC3T3 osteoblasts, and (iv) in mesenchymal cell types present in early callus tissue during fracture repair. Loss of function studies using siRNA knockdown of *Prmt1*, *Prmt4/Carm1* and *Prmt5* in MC3T3 cells indicate that each of these Prmt members has distinct roles during osteoblast differentiation: *Prmt1* loss enhances while *Prmt5* loss inhibits calcium deposition, while *Prmt4/Carm1* loss by itself is dispensable for calcium deposition in the extracellular matrix of differentiating osteoblasts. Gene expression analysis reveals that loss of *Prmt1*, *Pmrt4/Carm1* and *Prmt5* each modulates the expression of several but not all stage-specific mRNA biomarkers for osteoblast differentiation, although not proliferation. Finally, pharmacological inhibition with the Class I PRMT inhibitor GSK715 enhances calcium deposition in the extracellular matrix of MC3T3 cells. Collectively, these data establish that PRMT proteins have distinct effects on osteoblast differentiation.

Several technical limitations qualify our interpretations and conclusions. One major assay for the effects of PRMT loss of osteoblast differentiation is Alizarin Red staining which measures calcium deposition and not necessarily deposition of bone mineral (hydroxyapatite) in MC3T3 cells. While methods that are more definitive for hydroxyapatite (e.g., Von Kossa staining) could be applied, we consider that examination of definitive effects on bone mineralization will require in vivo studies that are beyond the scope of the present work. This study does not focus on recapitulating fidelity of bone-tissue like organization, but rather we use calcium deposition as a proxy for osteoblast differentiation to understand which PRMTs modulate osteoblastogenesis. Furthermore, our study includes only a single PRMT drug (GSK715) which exhibits non-specific negative effects at higher micromolar doses. Therefore, it will be important to survey additional PRMT drugs, similar to studies we performed to screen for EZH2 inhibitors (Galvan et al., 2021) before we can commit to an in-depth analysis of the transcriptome, epigenetic landscape and histone arginine methylome that are beyond the scope of the present work.

Our study shows that PRMT1, PRMT4/CARM1 and PRMT5 each have different functions in osteoblast maturation and/or expression of osteoblast related markers. Results obtained for PRMT5 are most easily interpretable. Loss of PRMT5 suppresses expression of multiple genes (i.e., Alpl, Ibsp, Phospho1) involved in mineralization and this finding corroborates reduced Alizarin Red staining upon siPRMT5 treatment. Our finding that PRMT1 inhibits osteoblast differentiation is in agreement with other studies indicating that PRMT1 promotes the undifferentiated state of cells (Goruppi et al., 2023). The results for PRMT4/CARM1 do not permit a straightforward interpretation, because siRNA depletion of this protein has no appreciable effects on osteoblast mineralization by Alizarin red staining. Yet, treatment of MC3T3 cells using GSK715 obliterates H3R17me2 levels (a mark that is catalyzed by PRMT4/CARM1) and appears to stimulate calcium deposition as a proxy for osteoblast differentiation. The latter may indicate that either siCARM1 effects are compensated by other PRMT proteins or that another Class I H3R17me2 methyl transferase beyond PRMT4/CARM1 is targeted by GSK715. Regardless of these two possibilities, this study provides the first evidence of the importance of H3 and H4 arginine methylation during osteogenesis.

Recent studies suggest that PRMT proteins also have molecular effects on the activity of the H3K27me3 transferase EZH2 that normally suppresses osteoblast differentiation (Dudakovic et al., 2016; Dudakovic et al., 2020). One solid study by the Nimer laboratory showed that PRMT5-mediated histone arginine methylation antagonizes and prevents the formation of H3K27me3 marks that are generated by EZH2 as part of the transcriptionally repressive polycomb complex PRC2 (Liu et al., 2020). Loss of PRMT5 results in a global rise of EZH2 dependent H3K27me3 marks because PRMT5 mediated methylation of H3 blocks subsequent methylation by PRC2 (Liu et al., 2020). Our finding that PRMT5 loss blocks osteoblast differentiation is consistent with this molecular mechanism. Loss of PRMT5 is predicted to prevent arginine methylation of H3 which is predicted to enhance H3K27me3 levels (Liu et al., 2020), and the latter has been associated with suppression of osteoblast differentiation. A second mechanism by which PRMT proteins can affect EZH2 activity is via direct methylation of EZH2 (at R342) by PRMT1, as has been suggested by another recent study (Li et al., 2020). This methylation step has been reported to stabilize EZH2 and increased EZH2 levels are predicted to block osteoblast differentiation (Li et al., 2020). Importantly, PRMT5 mediated increases in H3 arginine methylation and enhanced H3K27me3 levels are consistent with our observation that siRNA depletion of PRMT5 blocks osteogenesis. Similarly, the finding that PRMT1 depletion accelerates osteogenesis is in agreement with the model that loss of PRMT1 reduces EZH2 stability.

Our findings on the distinct functions of PRMT1, PRMT4/CARM1 and PRMT5 complement and extend previous studies that examined the contribution of PRMTs and their cognate epigenetic modifications in vitamin D3-dependent transcriptional regulation in rat osteosarcoma cells. One previous study showed that PRMT1 and PRMT4/CARM1 support the vitamin D3 (1,25(OH)_2_ D_3_) dependent upregulation of *CYP24A1* gene expression (Moena et al., 2020b). Examination of histone marks revealed that H4R3me2a and H3R17me2a and their corresponding writers PRMT1 and PRMT4, respectively, are recruited by vitamin D receptor (VDR) and its coactivator SRC-1/NCOA1. Both PRMTs are released when the VDR/SRC-1 complex is stimulated by (1,25(OH)_2_ D_3_). Similarly, when the *CYP24A1* gene promoter remains transcriptionally silent, the *CYP24A1* locus is enriched with both H3R3me2s marks and the corresponding methyl transferase PRMT5 (Moena et al., 2020b). Furthermore, PRMT5 and the SWI/SNF protein complex are involved in the regulation of 1,25(OH)_2_ D_3_ metabolism, which suggests that this enzyme may play a role in the maintenance of calcium homeostasis by vitamin D (Seth-Vollenweider et al., 2014). Taken together, the results from the current study and other reports (Li et al., 2017; Blanc and Richard, 2017) indicate that PRMTs control osteoblast-related gene expression.

In conclusion, our study shows that PRMT1, PRMT4/CARM1 and PRMT5 proteins each have different effects on osteoblast mineralization and/or expression of osteoblast-related differentiation markers. The mechanisms by which PRMT1 and PRMT5 control osteoblast differentiation may involve effects on the histone arginine methylome, as well as direct and indirect effects on EZH2, which is a known suppressor of osteogenesis. One translationally relevant finding from our study is that the Class I PRMT inhibitor GSK715 enhances osteoblast differentiation. This finding predicts that GSK715 may potentially have bone anabolic effects in mouse models for low bone mineral density, a prediction that will be tested in future studies.

## Funding

This publication was made possible through internal funding of 10.13039/501100010659Erasmus University Medical Center (to BCJvdE), the 10.13039/100000069National Institute of Arthritis and Musculoskeletal and Skin Diseases (R01 AR069049 to AvW & R01AR039588 to GSS), as well as the 10.13039/100009395CancerCare Manitoba Foundation (761020451 to JD).

## Authorship contributions

PD performed the majority of the experiments, while EAL contributed additional supporting data, reagents and experimental ideas. PD, EAL, JARG, MAM, JPTMvL, GSS, BCJvdE, JRD & AJvW provided principal conceptual contributions pertinent to the conclusions of this work. PD prepared the first draft of this manuscript, while EAL, JARG, MAM, JPTMvL, GSS, BCJvdE, JRD & AJvW each provided critical input and suggested edits. AJvW obtained the main source of funding for this study, while EAL, GSS, BCJvdE and JRD provided auxiliary funding.

## CRediT authorship contribution statement

**Parisa Dashti:** Conceptualization, Data curation, Formal analysis, Investigation, Methodology, Resources, Validation, Visualization, Writing – original draft, Writing – review & editing. **Eric A. Lewallen:** Conceptualization, Funding acquisition, Investigation, Writing – review & editing. **Jonathan A.R. Gordon:** Conceptualization, Writing – review & editing. **Martin A. Montecino:** Conceptualization, Writing - review & editing. **Johannes P.T.M. van Leeuwen:** Conceptualization, Project administration, Supervision, Writing – review & editing. **Gary S. Stein:** Conceptualization, Funding acquisition, Writing – review & editing. **Bram C.J. van der Eerden:** Conceptualization, Project administration, Supervision, Writing – review & editing. **James R. Davie:** Conceptualization, Funding acquisition, Writing – review & editing. **Andre J. van Wijnen:** Conceptualization, Data curation, Funding acquisition, Investigation, Resources, Supervision, Validation, Writing – review & editing.

## Declaration of competing interest

The authors have no conflicts of interest to declare. All co-authors have seen and agree with the contents of the manuscript and there is no financial interest to report.

## Data Availability

RNA-seq data are available on request and can be retrieved via links available in previous papers from our group.

## Appendix A Supplementary data

Supplementary Fig. 1: RNA-seq data from mesenchymal versus non-mesenchymal cell types in mouse callus during fracture repair. (A) Schematic illustration showing sorted mesenchymal and non-mesenchymal cells from mouse fracture callus at day 2 or day 6 after fracture (Matthews et al., 2014). Mesenchymal cells were marked using a tdTomato transgene that is recombined by a tamoxifen-inducible Cre-ERT2 recombinase controlled by the αSMA/ACTA2 promoter (Matthews et al., 2014). (B) RNA-seq data (in FPKM) show that Prmt1, Prmt4 and Prmt5 are preferentially expressed in mesenchymal cell types compared to non-mesenchymal cell types in callus tissue at day 2 after fracture in the fracture site. Due to technical limits in sourcing these sorted cells from fracture callus, each of the RNA-seq samples were analyzed as a single barcoded library from pooled callus samples; these data illustrate relative differences in PRMT expression in mesenchymal versus non-mesenchymal cells. Supplementary Fig. 2: *PRMT* family expression in human musculoskeletal tissues. RNA-seq data were retrieved from human musculoskeletal tissues including bone, cartilage, growth plate, muscle and adipose tissue, as previously reported (Liu et al., 2020). These data were obtained using human tissue samples from consenting adults undergoing orthopedic surgery or from the datasets from the GEO database that we have re-analyzed using the same bioinformatic pipeline to create a matrix of RNA-seq data across multiple human musculoskeletal tissues. *PRMT1* has highest expression in human bone, muscle, adipose, growth plate, and cartilage samples compared to other *PRMT* family members. *PRMT4/CARM1* and *PRMT5* are also expressed at high levels in human musculoskeletal tissues, specifically in muscle and cartilage (n = 6 samples, mean + STD). Supplementary Fig. 3: *PRMT* family expression in human mesenchymal stem/stromal cells. RNA-seq data shows that *PRMT1, PRMT4/CARM1,* and *PRMT5* expressions are higher than other *PRMT* members during very early stages of osteogenic differentiation in uncommitted human bone marrow derived mesenchymal stromal cells (BMSCs) that were cultured in osteogenic media. The line graphs show average expression values for each gene across multiple timepoints for two different adult human donors (n = 2 samples; Lonza). Supplementary Fig. 4: *PRMT* knock-down has no significant effect on proliferation markers during osteoblast differentiation. RT-qPCR analysis shows *Cyclin B2 /Ccnb2* and *Mki67* mRNA expression levels are not notably altered upon: (A) *Prmt1* knock-down, (B) *Prmt4/Carm1* knock-down, or (C) *Prmt5* knock-down over the 15 day course of osteoblast differentiation (n = 3, mean ± STD; *=p < 0.01, **=p < 0.001, and ***=p < 0.0001). Supplementary Fig. 5: *Prmt4/Carm1 loss* inhibits *Runx2* mRNA expression level in MC3T3 cells. RT-qPCR analysis for osteoblast transcription factors (*Runx2*, *Osterix /Sp7*, and *Dlx5*) shows that (A) expression of select transcription factors does not change during osteoblast differentiation upon *Prmt1* knockdown, while (B) *Prmt4/Carm1* knockdown decreases *Runx2* expression in early stages of osteoblast differentiation (day 6), while other transcription factors do not show appreciable alterations in expression. (C) *Prmt5* knockdown blocks expression of *Osterix/Sp7* mRNA during a late stage (day 10) of osteoblast differentiation in MC3T3 cells (n = 3, mean ± STD; **=p < 0.01, ***=p < 0.001, and ****=p < 0.0001). Supplementary Fig. 6: Assessment of osteoblast epigenetic regulators upon *Prmt1*, *Prmt4/Carm1*, and *Prmt5* knock-down in MC3T3 cells. RT-qPCR analysis shows epigenetic regulator markers (*Ezh1*, *Ezh2*, *Brd2*, and *Brd4*) mRNA expression levels barely change upon (A) *Prmt1* knock-down (B) *Prmt4/Carm1* knock-down (C) *Prmt5* knock-down over the osteoblast differentiation in 15 days. (n = 3, mean ± STD; ***=p < 0.001). Supplementary Fig. 7: Quantification of Alizarin Red staining of MC3T3 cell cultures. Alizarin Red staining of differentiated MC3T3 cells (at day 28) after treatment with 1 dose (day 0 to day 3) (A) or 2 doses of GSK715 (day 0 to day 6) (B) at a range of concentrations from 1.4 to 50 μM (x-axis). The surface areas of the culture plates that were stained with Alizarin Red (see Fig. 8) were measured by ImageJ in arbitrary densitometric units (A). Average densitometric values (y-axis) of triplicate samples (blue dots) were fitted to a polynomial curve (blue dotted line). The relative fit for the 1× and 2× dose response curves is characterized by the R^2^ values 0.2185 and 0.5589. Because semi-log curves do not permit visualization of the value zero, the average value for vehicle (0 μM GSK715) is represented by horizontal black dashed line. For reference, we also provided an additional horizontal red dashed line for a representative concentration of GSK715 (2.9 μM) as a visual aid to show intersection with the y-axis compared to vehicle control. Image 1
