# Supplementary material for: Protein arginine methyltransferases PRMT1, PRMT4/CARM1 and PRMT5 have distinct functions in control of osteoblast differentiation
Source: Bone Rep. 2023 Jul 25;19:101704. doi: 10.1016/j.bonr.2023.101704 (PMC10430181; doi:10.1016/j.bonr.2023.101704)
Supplement: Supplementary file 1 — Supplementary Fig. 1: RNA-seq data from mesenchymal versus non-mesenchymal cell types in mouse callus during fracture repair. (A) Schematic illustration showing sorted mesenchymal and non-mesenchymal cells from mouse fracture callus at day 2 or day 6 after fracture (Matthews et al., 2014). Mesenchymal cells were marked using a tdTomato transgene that is recombined by a tamoxifen-inducible Cre-ERT2 recombinase controlled by the αSMA/ACTA2 promoter (Matthews et al., 2014). (B) RNA-seq data (in FPKM) show that Prmt1, Prmt4 and Prmt5 are preferentially expressed in mesenchymal cell types compared to non-mesenchymal cell types in callus tissue at day 2 after fracture in the fracture site. Due to technical limits in sourcing these sorted cells from fracture callus, each of the RNA-seq samples were analyzed as a single barcoded library from pooled callus samples; these data illustrate relative differences in PRMT expression in mesenchymal versus non-mesenchymal cells. Supplementary Fig. 2: PRMT family expression in human musculoskeletal tissues. RNA-seq data were retrieved from human musculoskeletal tissues including bone, cartilage, growth plate, muscle and adipose tissue, as previously reported (Liu et al., 2020). These data were obtained using human tissue samples from consenting adults undergoing orthopedic surgery or from the datasets from the GEO database that we have re-analyzed using the same bioinformatic pipeline to create a matrix of RNA-seq data across multiple human musculoskeletal tissues. PRMT1 has highest expression in human bone, muscle, adipose, growth plate, and cartilage samples compared to other PRMT family members. PRMT4/CARM1 and PRMT5 are also expressed at high levels in human musculoskeletal tissues, specifically in muscle and cartilage (n = 6 samples, mean + STD). Supplementary Fig. 3: PRMT family expression in human mesenchymal stem/stromal cells. RNA-seq data shows that PRMT1, PRMT4/CARM1, and PRMT5 expressions are higher than other PRMT members [file mmc1.pptx]

## Slide 1
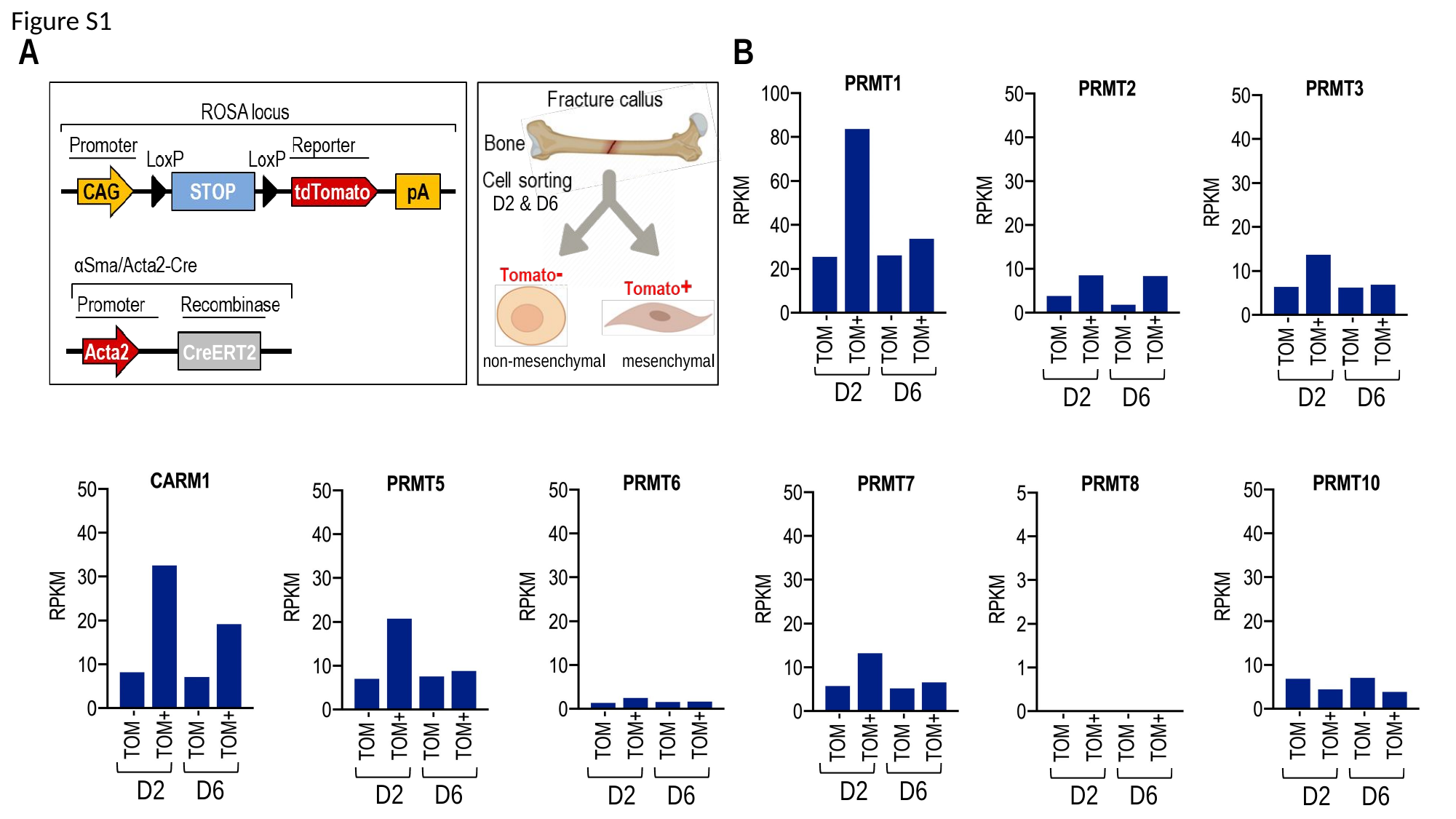

Figure S1
A
B
D2 & D6
CreERT2
non-mesenchymal
mesenchymal
D2 D6
D2 D6
D2 D6
D2 D6
D2 D6
D2 D6
D2 D6
D2 D6
D2 D6

## Slide 2
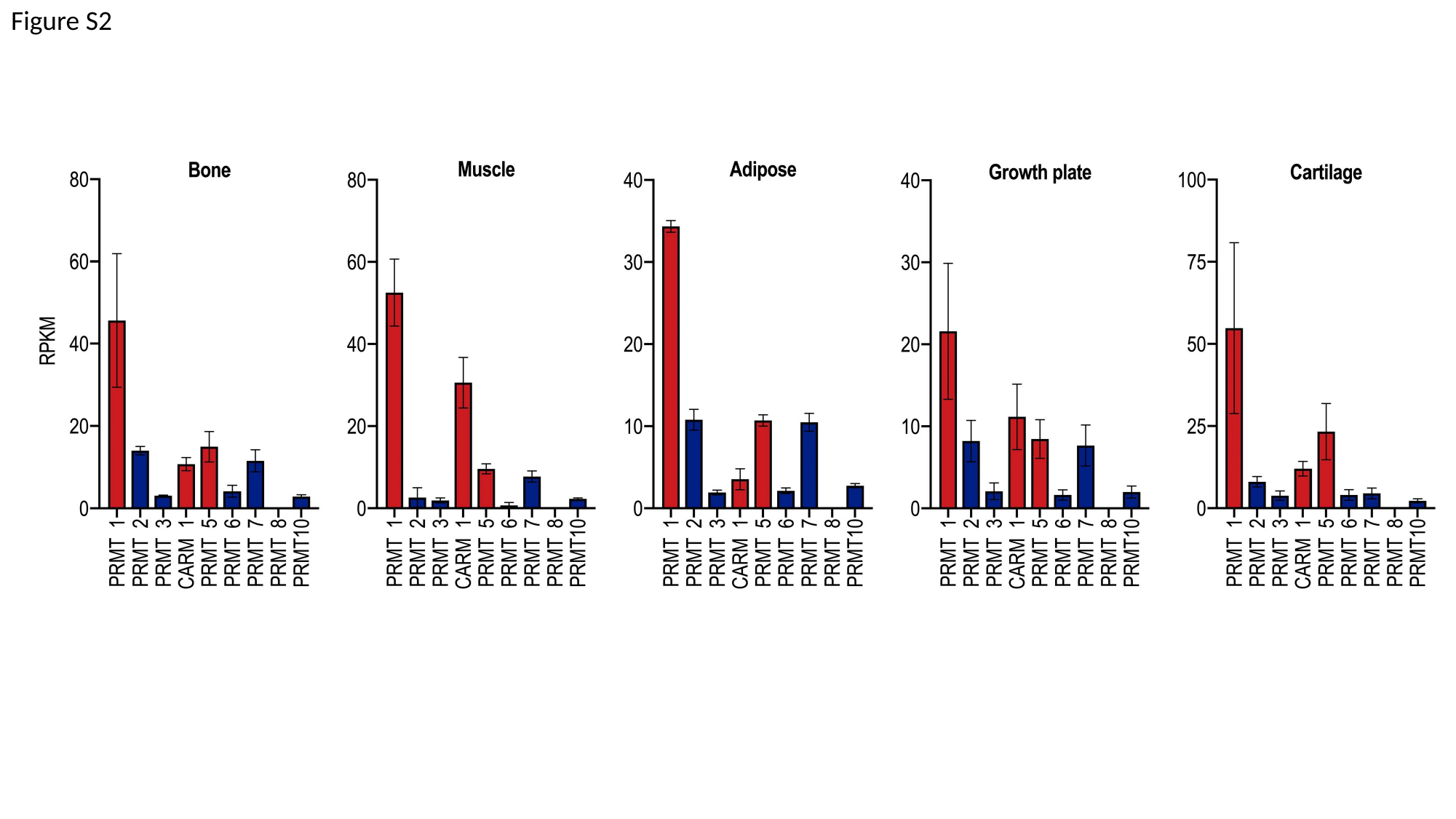

Figure S2

## Slide 3
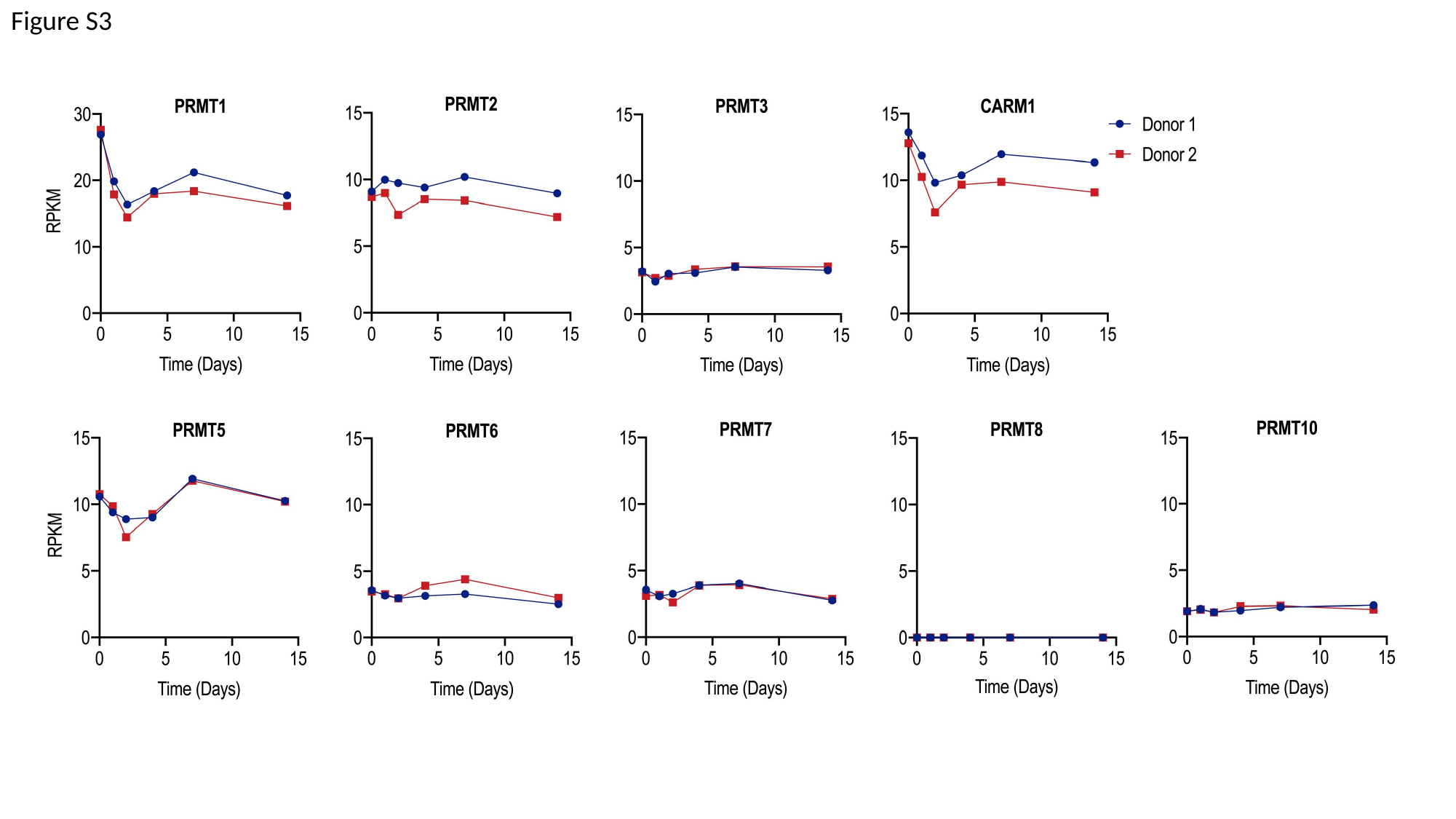

Figure S3

## Slide 4
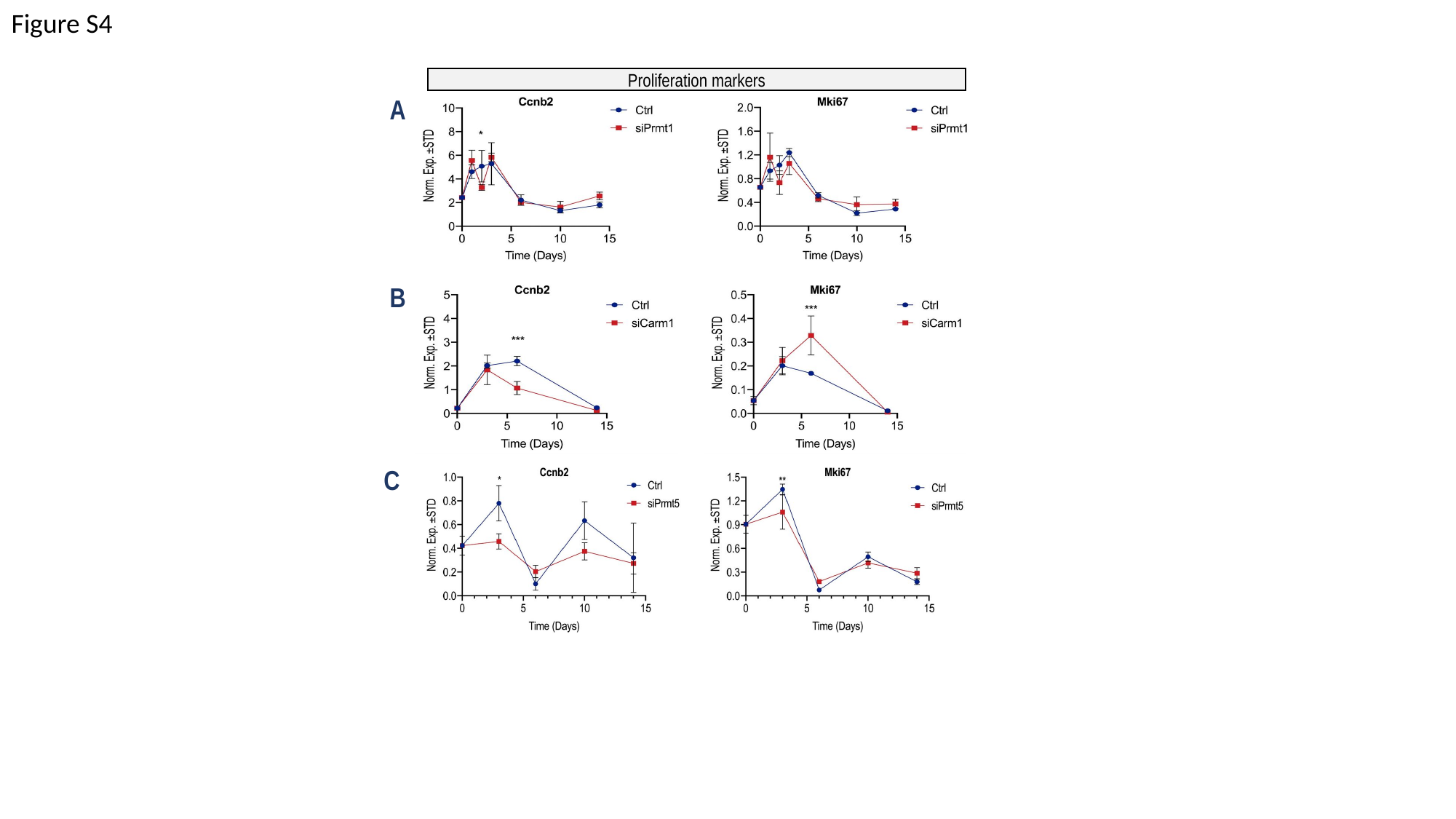

Figure S4
Proliferation markers
A
B
C

## Slide 5
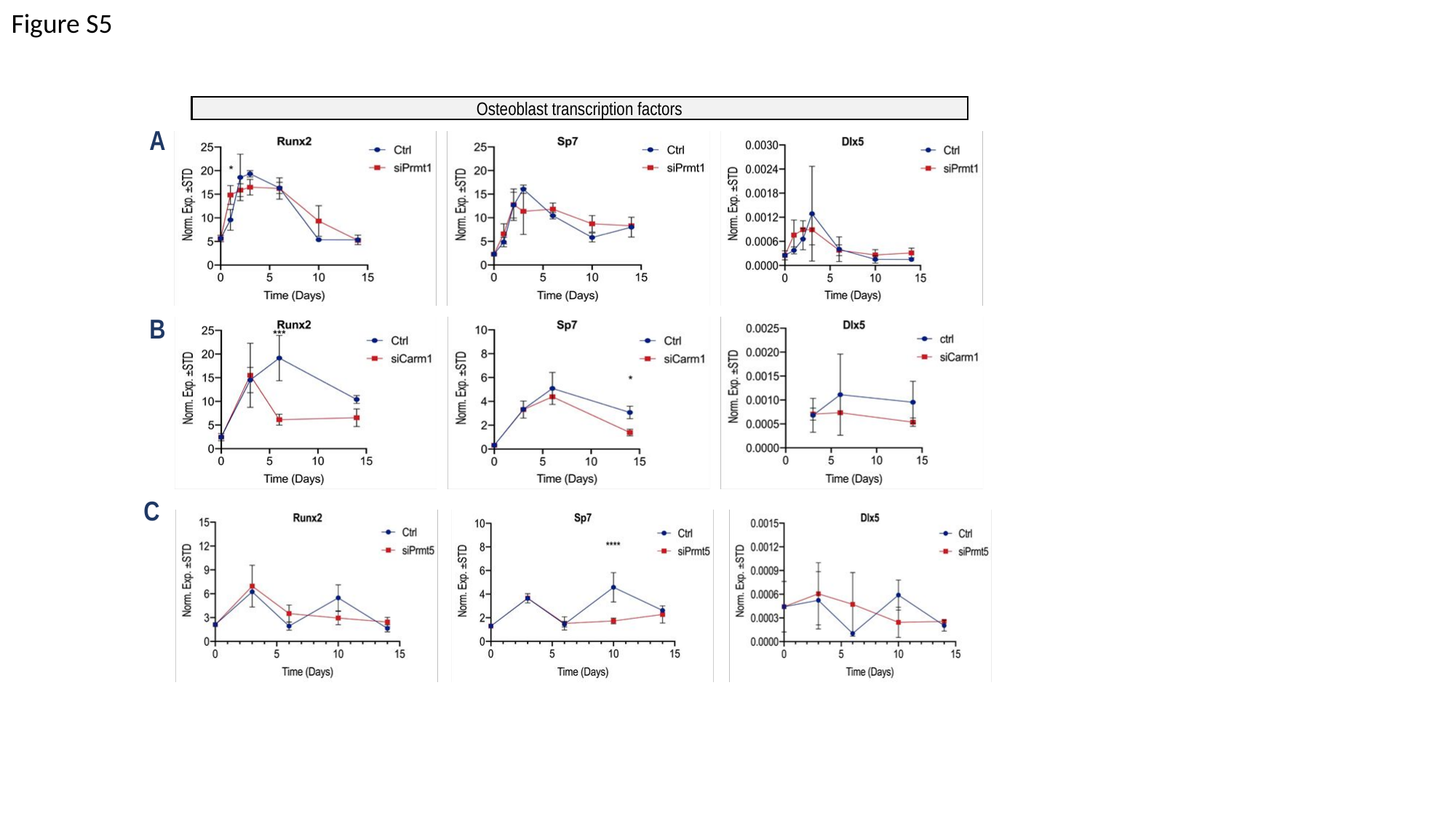

Figure S5
Osteoblast transcription factors
A
B
C

## Slide 6
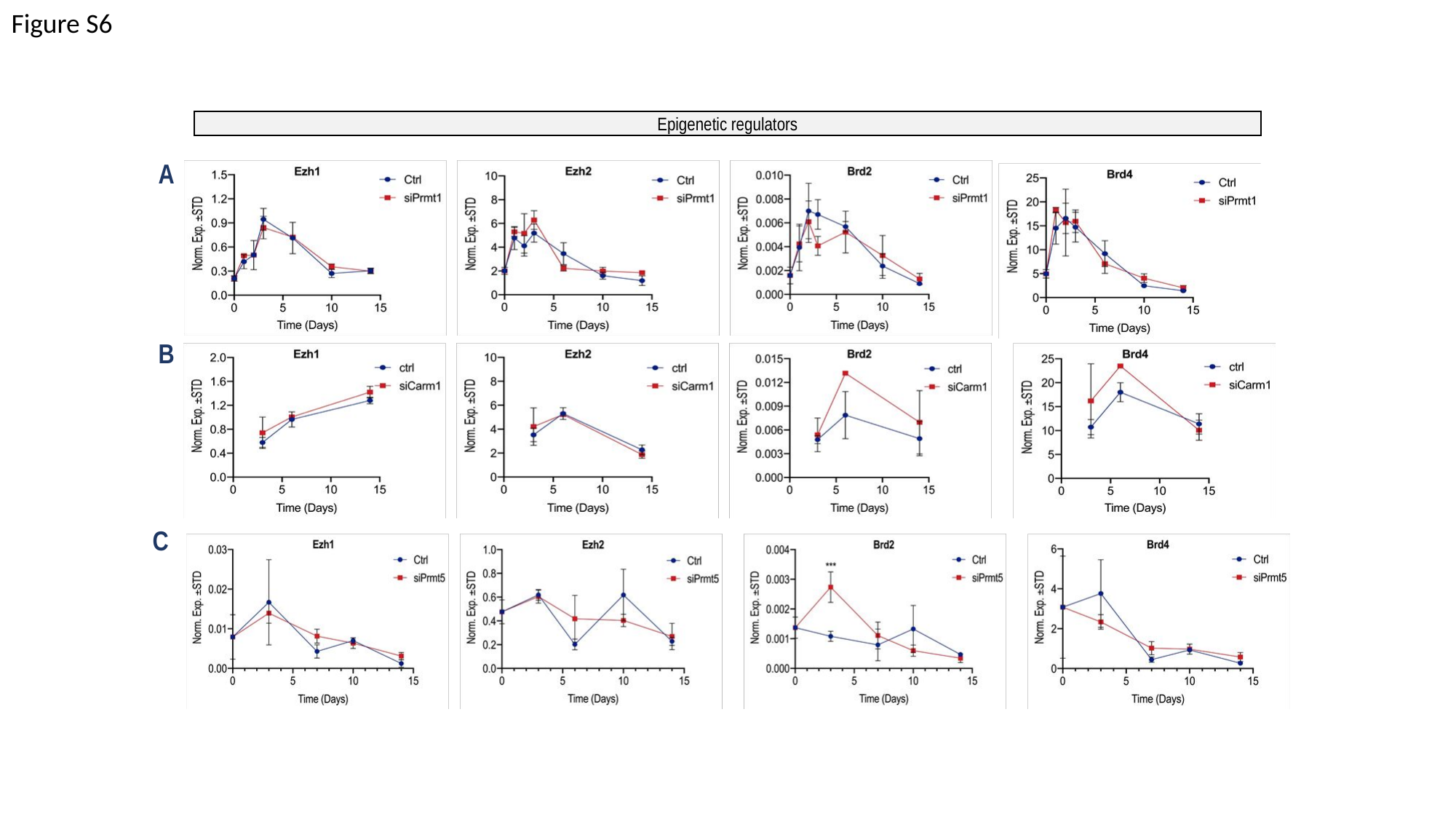

Figure S6
Epigenetic regulators
A
B
C

## Slide 7
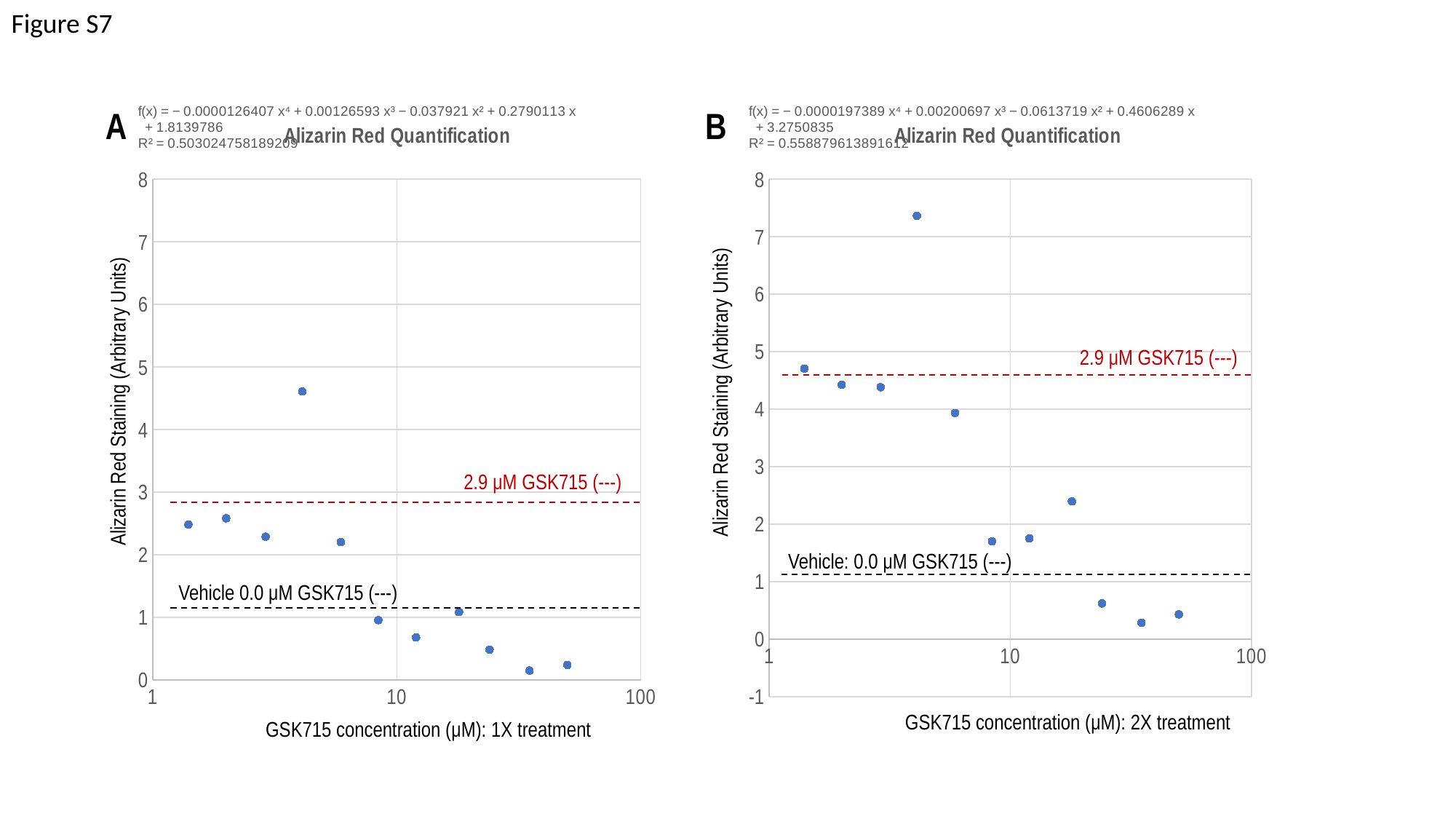

Figure S7
A
B
### Chart: Alizarin Red Quantification
| Category | |
|---|---|
### Chart: Alizarin Red Quantification
| Category | |
|---|---|2.9 μM GSK715 (---)
Alizarin Red Staining (Arbitrary Units)
Alizarin Red Staining (Arbitrary Units)
2.9 μM GSK715 (---)
Vehicle: 0.0 μM GSK715 (---)
Vehicle 0.0 μM GSK715 (---)
GSK715 concentration (μM): 2X treatment
GSK715 concentration (μM): 1X treatment
